# Supplementary material for: Content-rich biological network constructed by mining PubMed abstracts
Source: BMC Bioinformatics. 2004 Oct 8;5:147. doi: 10.1186/1471-2105-5-147 (PMC528731; doi:10.1186/1471-2105-5-147)
Supplement: Additional File 5 — The original Chilibot query results of the term "long-term potentiation (LTP)" and 22 other terms, limiting the latest references analyzed to the years 1990, 1995, 2000, and 2004. [file 1471-2105-5-147-S5.bz2 › chilibotAdditionalFile5/ltp2004/html/TRKB_SYNAPTOPHYSIN.html]

 


 **TRKB** and **SYNAPTOPHYSIN** 
  
Found 11 abstracts in PubMed,  **11 abstracts were retrieved and analyzed**.  


---

 Search Google  |
 PDF files only 
|  EDU domain only 

---

**Interactive relationship** (e.g. stimulation, inhibition, etc)

- We find that BDNF but not NGF rapidly upregulates levels of  **synaptophysin** , tau and c Fos correlating with expression of the neurotrophin receptor  **TrkB** .  Ref: 9185679 Neurosci Lett, 1997
- A comparison of  **trkB**  with p75, which is expressed in afferent and efferent fibers, and GAP 43 and  **synaptophysin** , which are expressed in efferent fibers, revealed a time space correlation of  **trkB**  receptor expression in hair cells with the rearrangement of their innervation.  Ref: 8593664 Cell Tissue Res, 1996

**Parallel relationship** (e.g. studied together, co-existance, homology, etc.)

- Brain derived neurotrophic factor induces a rapid upregulation of  **synaptophysin**  and tau proteins via the neurotrophin receptor  **TrkB**  in rat cerebellar granule cells.We have examined the effects of neurotrophins brain derived neurotrophic factor and nerve growth factor on the expression of the maturation specific proteins  **synaptophysin**  and tau, and the growth associated protein 43 in cerebellar granule cells.  Ref: 9185679 Neurosci Lett, 1997
- In addition to routine histological examinations, immunohistochemistry was used to evaluate the expression of neurotrophin receptors TrkA,  **TrkB**  and of neuronal differentiation markers such as neuron specific enolase, neurofilament,  **synaptophysin** , and chromogranin A.  Ref: 9625303 Clin NeuropatholClin Neuropathol, 1997
- To test the hypothesis whether a failure to express neurotrophins or a neurotrophin receptor might underlie the pathology observed in mutant mice with degeneration of regionally distinct subpopulations of neurons, the expression of BDNF, NT 3,  **TrkB** , TrkC and  **synaptophysin**  mRNA was examined in the cerebellum of mutant lurcher lc and weaver wv wv wv mice.  Ref: 9733904 Brain Res Dev Brain Res, 1998
